# Monte Carlo modeling and phantom studies show Cherenkov emission per unit dose during total skin electron therapy is a function of tissue optical properties

**DOI:** 10.1002/mp.70521

**Published:** 2026-06-08

**Authors:** Yifeng Zhu, Yi Hong Ong, Brook K. Byrd, Daniel A. Alexander, Madelyn Johnson, Dennis Sourvanos, Timothy C. Zhu

**Affiliations:** ^1^ Department of Radiation Oncology Perelman School of Medicine University of Pennsylvania Philadelphia Pennsylvania USA; ^2^ Department of Periodontics School of Dental Medicine University of Pennsylvania Philadelphia Pennsylvania USA

**Keywords:** Cherenkov emission, light propagation, MC simulation, tissue optical properties, TSET

## Abstract

**Background:**

Cherenkov imaging provides a noninvasive approach to visualize total skin electron therapy (TSET) radiation dose deposition on patient. Accurate conversion of Cherenkov intensity to radiation dose is necessary for in vivo dosimetry to assess the spatial dose distribution in TSET. Studies have shown a linear correlation between Cherenkov intensity and absorbed dose, but the effect of tissue optical properties on the Cherenkov emission per dose is not well understood.

**Purpose:**

This work uses Monte Carlo simulations and experiments to assess how tissue optical properties affect the Cherenkov emission per dose detected during TSET.

**Methods:**

Monte Carlo modeling was used to simulate Cherenkov generation during total skin electron therapy and quantify the effect of tissue optical properties on the detected Cherenkov emission. The study examined a clinically relevant range of absorption coefficients (0.01–1 cm^−1^) and reduced scattering coefficients (2–40 cm^−1^) at 665 nm. The effect of tissue optical properties, depth of origin and the angular distribution of Cherenkov emission on tissue surface were systematically evaluated. The Monte Carlo results are compared to measurements for a series of solid phantoms. An analytical function is proposed to fit the optical properties dependence (µ_a_ and µ_s_’) of Cherenkov emission for tissue.

**Results:**

Simulation results show that Cherenkov emission decreases with increasing tissue absorption and effective attenuation coefficients but increases then decreases with tissue scattering coefficients. 80% of the surface‐detected emission originated from superficial layers 0.17‐ 2.0 cm beneath the surface. Angle‐specific generation of Cherenkov radiation in tissue has not resulted in preferential exiting angle as the propagation directions of most Cherenkov photons are randomized prior to reaching the surface. Monte Carlo simulation (max dev 0.23%) agrees with experiments to within a standard (maximum) % deviation of 2.8% (7.6%).

**Conclusion:**

Our findings indicate that tissue optical properties exert a substantial influence on the surface Cherenkov emission. The optical properties dependence of Cherenkov emission per dose can be expressed as a two‐dimensional function of µ_a_ and µ_s_’. An analytical expression is presented. Monte Carlo simulation agrees with experiments for TSE electrons used in a series of tissue simulating phantoms.

## 1 INTRODUCTION

Total skin electron therapy (TSET) involves treating the whole skin through electron beams generated by a linear accelerator. Cherenkov photons are created when high‐energy electrons travel through patient's body at velocities exceeding the phase speed of light within biological tissues.^1^, ^2^ Imaging of Cherenkov photons emitted from tissue surface has been demonstrated using specialized camera system to visualize patient dose deposition in real time.^3^, ^4^, ^5^ The implementation of Cherenkov imaging would offer a means to detect abnormalities in treatments in real time with excellent resolution. A linear relationship was found between locally deposited dose and Cherenkov emission from a slab phantom made of opaque water equivalent plastic while irradiating it with same beam energies.^6^ This suggests the potential use of Cherenkov emission as a tool of in vivo dosimetry to characterize spatial dose distribution for quality assurance during TSET. However, the linearity between dose and Cherenkov emission from tissue could be affected by tissue optical properties due to absorption and scattering of Cherenkov photons when interacting with tissues.^7^ In clinical settings, variations in skin type, pigmentation, or discoloration introduce spatial heterogeneity in tissue optical properties, which is frequently observed among patients. Different attenuation of Cherenkov emission could easily be mistaken for non‐uniform dose deposition if spatial heterogeneities in tissue optical properties are not accounted for carefully. Moreover, human tissue optical properties exhibit substantial inter‐patient variability, making it impossible to apply a universal equation to convert Cherenkov intensity into dose. The Cherenkov‐to‐dose conversion factor can vary both within an individual and across different patients. Furthermore, the depth and volume of tissue in which Cherenkov photons are generated can also vary substantially depending on tissue optical properties.

Prior Monte Carlo studies have investigated Cherenkov photons transport in tissue using effective bulk optical properties. Glaser et al.^8^ performed Monte Carlo simulations of Cherenkov radiation transport in soft tissue to in vivo Cherenkov imaging under an effective homogeneous depth assumption, but did not derive a generalized quantitative relationship between Cherenkov emission and tissue optical properties. More recently, Parks et al.^9^ reported a hybrid Monte Carlo model for efficient estimation of tissue Cherenkov emission under varying beam size, energy, and incidence, but the study remained a forward model of emitted spectra rather than a framework relating Cherenkov emission directly to µ_a_ and µ_s_’. Multilayer skin geometries have also been used in Monte Carlo studies,^1^, ^10^ but the added dimensionality makes derivation of a practical generalized relationship between Cherenkov emission and tissue optical properties difficult. A quantitative description of Cherenkov emission per dose as a function of tissue optical properties over a clinically relevant range for TSET is still not available.

In this study, Monte Carlo simulations were used to investigate how tissue optical properties affect Cherenkov emission per dose detected at the surface. The emission spectrum was modeled from 400 to 900 nm across a range of clinically relevant optical properties at 665 nm (µ_a_ = 0.01 – 1 cm^−1^, µ_s_’ = 2 – 40 cm^−1^) based on reported in‐vivo tissue optical properties.^11^ For each wavelength, absorption coefficients were derived from the absorption spectra of oxy‐ and deoxyhemoglobin, while reduced scattering coefficients (µ_s_' = µ_s_(1‐g)) were estimated using Mie scattering theory, µ_s_’(λ) = Aλ^−b^. The effect of tissue optical properties on the emission spectrum, angular distribution, and depth of origin of Cherenkov photons escaping the tissue surface were systematically investigated in an earlier study,^7^ unfortunately the results were incorrect due to a bug of expressing the voxel dimension in unit of mean‐free path rather than physical depth in the original MC code. Our current MC study has corrected these bugs which resulted in completely different tissue optical properties dependence, e.g., the tissue optical properties dependence of total Cherenkov emission in the original paper^7^ was a monotonic function of *R_d_*, while the current results are a two‐dimensional function of *R_d_* and µ_s_’. The new MC results are validated by a modified independent open source code (MCmatlab^12^) as well as experiments.

## 2 MATERIALS AND METHODS

**FIGURE 1:**
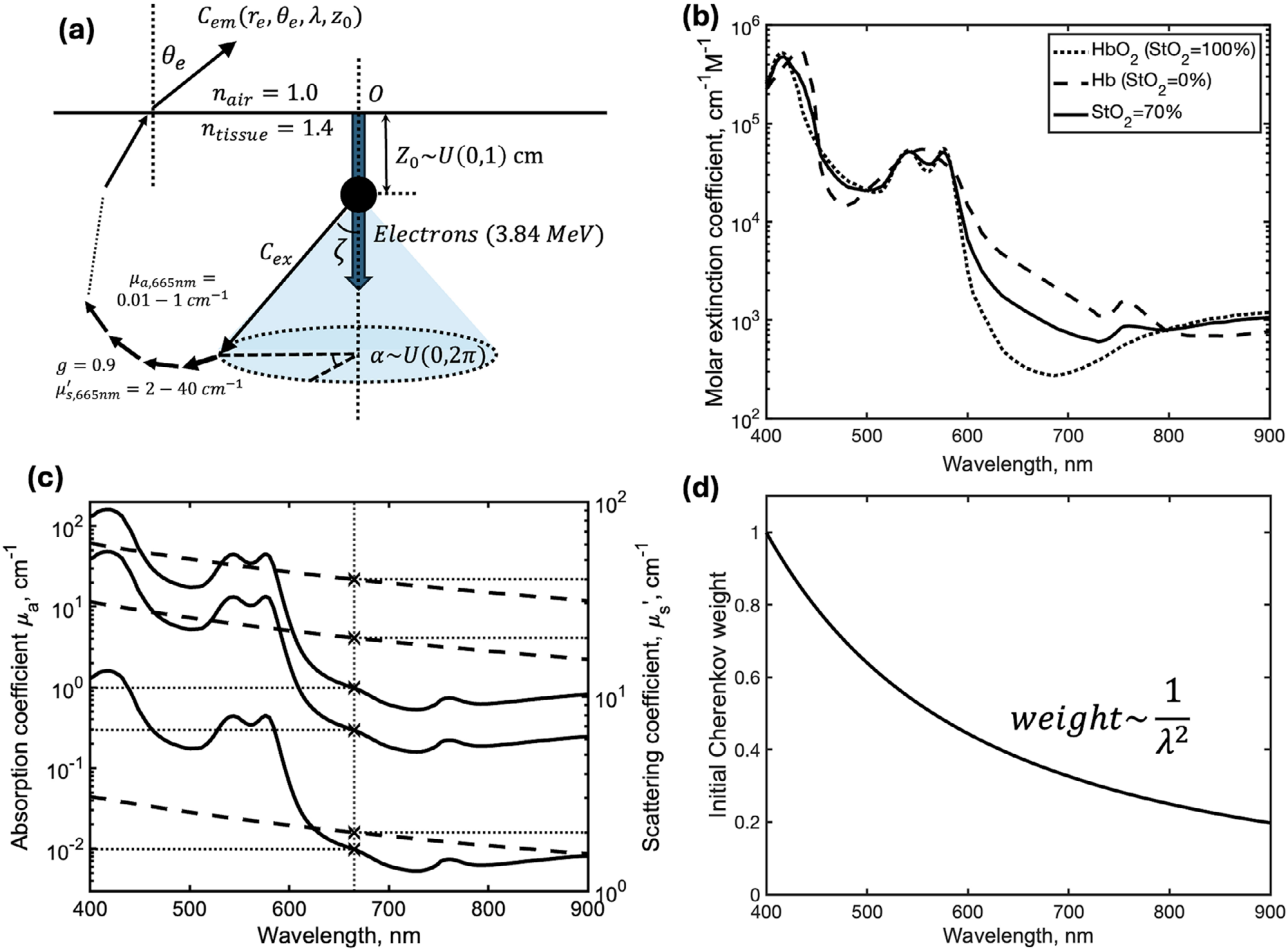
(a) Setup geometry for Monte‐Carlo simulation for a semi‐infinite turbid medium. (b) Extinction coefficient spectra of oxygenated and deoxygenated hemoglobin over wavelength range between 400–900 nm. Solid line represents molar extinction coefficient spectrum at 70% tissue oxygen saturation (StO_2_). (c) Absorption coefficients and reduced scattering coefficients over wavelength range between 400 to 900 nm. Solid lines are the absorption spectra for three conditions: µ_a_ = 0.01, 0.3 and 1 cm^−1^ at 665 nm. Dashed lines are the scattering spectra used in MC simulations for µ_s_’ = 2, 20, 40 cm^−1^ at 665 nm. (d) Wavelength dependence of initial Cherenkov photon weight over the wavelength between 400–900 nm.

The Monte Carlo algorithm used here was written in MATLAB (The MathWorks Inc., Natick, MA.) as described previously^13^, ^14^, ^15^ and modified to be consistent with the results of modified MCmatlab.^12^ The simulations were performed in a semi‐infinite medium with uniform optical properties, as illustrated in Figure 1a. The anisotropy factor (g) was set to 0.9^16^ and the refraction mismatch n was set to 1.4^17^ (n_air_ = 1.0 and n_tissue_ = 1.4). Cherenkov photons were generated uniformly along a cone aligned with the central axis of electron propagation. The initial angle of the Cherenkov photon was determined from the electron energy according to:^18^

(1) $$cos\zeta =\frac{1}{\beta n}$$

where $\beta =\sqrt{1-{\left(\frac{mc^{2}}{E+mc^{2}}\right)}^{2}}$. The incident electrons were simplified to be uniformly distributed from the surface (*z*_0_ = 0 cm) to 1 cm depth, with the energy of 3.84 MeV representing the mean energy of clinical 6MeV TSET beams. For each simulation, ten million photons were generated with a polar angle (ζ) of 44° (derived from Eq.1) and an azimuthal angles (α) uniformly distributed between 0 and 2π. The wavelength range was 400–900 nm with 50 nm resolution. The initial depth (z_0_) was sampled uniformly distributed from 0 to 1 cm, as contributions from Cherenkov photons originating beyond this depth are negligible. Assuming 0 to 1 cm uniform distribution is closer to the actual depth dose distribution for 3.84 MeV electron beam than assuming 0 to 2 cm uniform distribution as used in the original study.^7^

Tissue optical properties (µ_a_ and µ_s_’) were derived from the spectra as shown in Figure 1b. Considering skin penetration depth for different wavelength light,^19^ oxyhemoglobin (HbO_2_) and deoxyhemoglobin (Hb) were considered the dominant absorbers and melanin absorption is ignored in this study. Absorption coefficients were calculated from the molar extinction spectra of HbO_2_ ($\varepsilon_{HbO_{2}}$) and Hb ($\varepsilon_{Hb}$):^20^

(2) $$\mu_{a,tissue}=THC\cdot \left(StO_{2}\cdot \varepsilon_{HbO_{2}}+\left(1-StO_{2}\right)\cdot \varepsilon_{Hb}\right)$$

where THC is the total hemoglobin concentration (THC = [HbO_2_] + [Hb]) and StO_2_ is the tissue oxygen saturation ($\mathrm{St}O_{2}=\left[\mathrm{Hb}O_{2}\right]/\left(\left[\mathrm{Hb}O_{2}\right]+\left[\mathrm{Hb}\right]\right)\times 100\%$), which was assumed to be 70%. To generate different optical properties, the spectrum is rescaled at 665 nm as shown in Figure 1c. The simulated tissue absorption at 665 nm ranged from 0.01 to 1 cm^−1^, corresponding to a THC of 42 to 420 µM. The reduced scattering spectrum was calculated using Mie scattering theory, *µ_s_’*(*λ*) = *Aλ^−b^*, where *b* was 0.838 as reported by Jacques^21^ for skin. The simulated reduced scattering coefficients ranged from 2–40 cm^−^^1^ at 665 nm and the corresponding *A* values were between 464.0–9280.8 cm^−1^, respectively. 42 combinations of different µ_a_ and µ_s_’ at 665 nm are used in these simulations with the whole range of 0.01–1 cm^−1^ and 2–40 cm^−1^, respectively (see Table 1).

**TABLE 1:** Monte Carlo simulation percentage statistical uncertainties (%) of total Cherenkov emission IChe, total, calculated by 1/$\sqrt{N}$, where *N* is the total photon numbers. The std for the entire phantom $\sqrt{\sum \sigma^{2}/\left(n-1\right)}$ is 0.14%.

µ_a_ / µ_s_'	2 cm^−1^	5 cm^−1^	10 cm^−1^	15 cm^−1^	20 cm^−1^	30 cm^−1^	40 cm^−1^
0.01 cm^−1^	0.05	0.05	0.05	0.05	0.06	0.06	0.06
0.1 cm^−1^	0.09	0.08	0.08	0.09	0.09	0.09	0.10
0.3 cm^−1^	0.13	0.12	0.12	0.12	0.12	0.13	0.13
0.5 cm^−1^	0.16	0.14	0.14	0.14	0.15	0.15	0.16
0.7 cm^−1^	0.19	0.17	0.16	0.16	0.17	0.17	0.18
1 cm^−1^	0.23	0.20	0.19	0.19	0.19	0.20	0.20

The initial weight of each photon was determined by λ^−2^ relationship,^22^, ^23^ as shown in Figure 1d. After being launched, each Cherenkov photon was tracked through multiple scattering events until it either exited the medium or its weight dropped below a threshold weight, initiating a Russian roulette process in which it survived with 10% probability (weight increased tenfold) or was terminated with 90% probability. Following each step, the photon weight was reduced by a factor of 1‐*a’*, where ($a^{\prime }=\mu_{s}^{\prime }/\left(\mu_{a}+\mu_{s}^{\prime }\right)$) and a new scattering angle was resampled according to the medium's optical property. Fresnel reflectance for unpolarized light was applied to account for specular reflection at the air‐tissue boundary. The escaped fractional Cherenkov photons (C_em_) were quantified with respect to lateral radius (*r*_e_), exiting angle (*θ*_e_), and wavelength (λ). Each photon carried a tag of its depth of origin (z_0_) which will be used to evaluate the depth of radiation dose corresponding to the detected Cherenkov intensity. A pencil irradiation beam approach was used in the Monte Carlo algorithm to simulate the generation of Cherenkov photons in tissue. The reciprocity theorem,^24^ which states that “the diffuse reflected photons escaping surface for a circular radiation field of radius *R* is equivalent to that for a pencil beam distribution on the central‐axis and scoring the escaped photons up to a circular area of radius *R”*, was used to calculate for Cherenkov emission for a broad circular incident electron beam.

To assess the effect of superficial melanin absorption, an additional two‐layer skin model was evaluated, consisting of a 100 µm epidermal layer^25^ over bulk tissue with the optical properties obtained from literature^26^: $\mu_{a,melanin}=f_{v}\cdot 519\cdot {\left(\frac{\lambda }{500\;\mathrm{nm}}\right)}^{-3}\;{\mathrm{cm}}^{-1}$, where the melanosome volume fraction *f_v_* was set to 0.02, 0.15, 0.30 to represent different skin types: light skinned Caucasians (µ_a,665 nm_ = 4.41 cm^−1^), well‐tanned Caucasians (µ_a,665 nm_ = 33.09 cm^−1^), and darkly pigmented Africans (µ_a,665 nm_ = 66.18 cm^−1^), respectively.^27^, ^28^ For the MC simulations, the melanin layer was modelled as an additional superficial absorber, while the tissue scattering properties were kept the same as the underlying tissue.^26^, ^29^ A broadband white‐light pencil beam source was used, and the diffuse reflectance spectrum (DRS), *R_d_*, was recorded over source‐detector (S‐D) separations of 0.14–0.87 (0.14, 0.20, 0.28, 0.34, 0.41, 0.48, 0.57, 0.67, 0.87) cm.^30^ The resulting spectra were analysed using a hybrid‐P3 approximation^31^ fitting approach that can more accurately fit the diffuse approximation in semi‐infinite turbid media. We opt to use component fitting and our fitting formula includes only two components (Oxy‐ and deoxy‐ hemoglobin, see Figure 1). To greatly improve the agreement between the fit and DRS raw data when some components (e.g., melanin) were missing, Fourier components are added in the fitting framework^32^ to distribute the error uniformly over the entire spectral range. This is the same fitting algorithm we use to analyze all patient skin DRS data to obtain the equivalent bulk tissue optical properties using the single‐layer model. Cherenkov simulations are then performed using the fitted absorption and scattering spectrum. The results were compared with those from the direct two‐layer simulation with the melanin layer to assess the effect of superficial melanin on the predicted Cherenkov emission.

**FIGURE 2:**
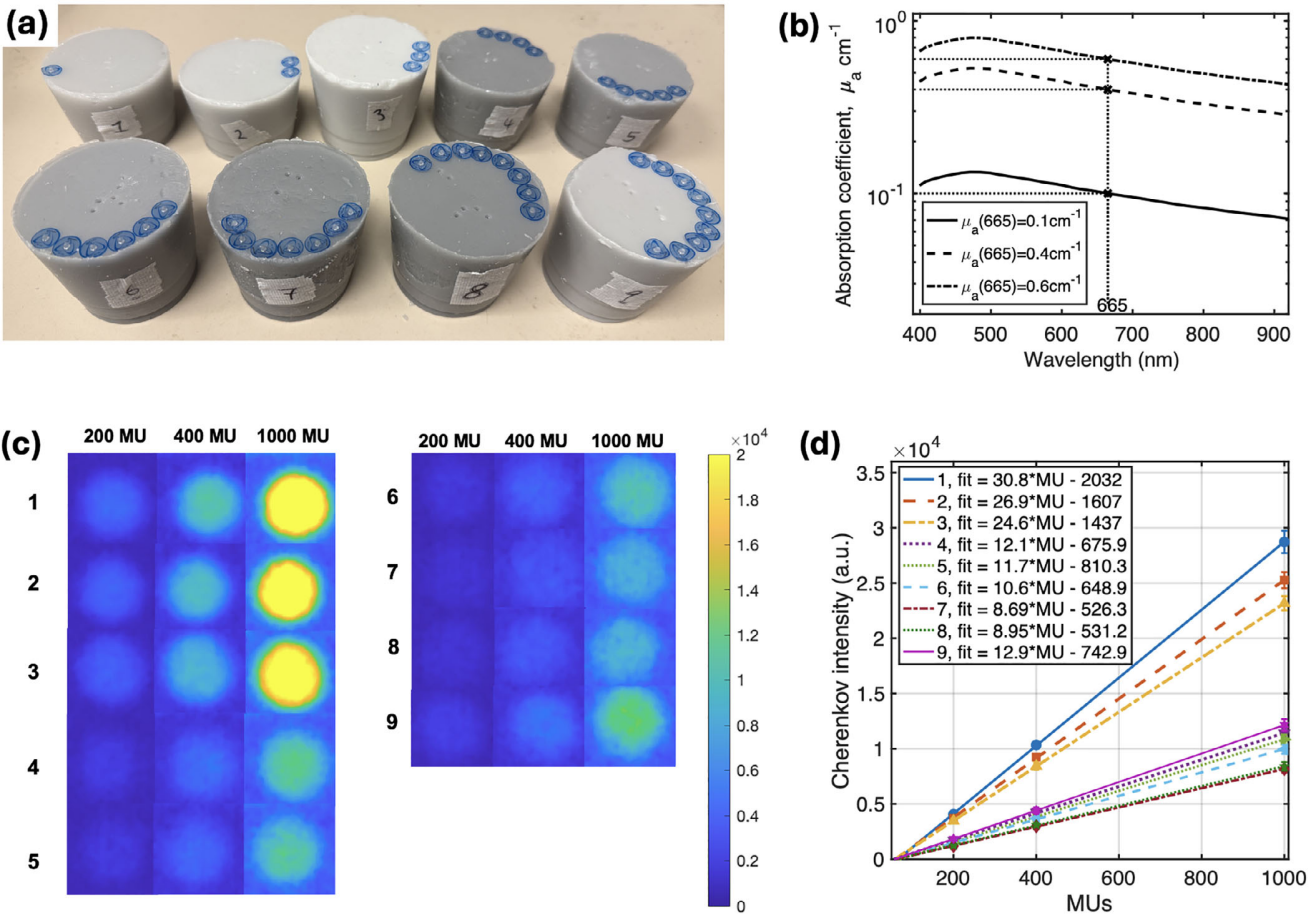
(a) Picture of 9 solid silicone gel phantoms made of carbon powder as absorber and TiO_2_ as scatterer. (b) Absorption coefficient spectra of carbon for 𝜇_𝑎_ = 0.1, 0.4, and 0.6 cm^−^^1^ at 665 nm. (c) Acquired Cherenkov images for 9 phantoms irradiated with HDTSE 6MeV electron beam at 200, 400 and 1000 MU at SSD 500 cm. (d) Cherenkov intensity versus MU for 9 phantoms (symbols) with different tissue optical properties (see Table 3). The lines are least squared linear fits.

To validate the dependence on tissue optical properties, we performed experiments with a set of solid phantoms of varying absorption and reduced scattering coefficients (Figure 2a). These standard tissue‐simulating phantoms, commonly used in the biomedical field,^33^ were made of silicone gel mixed with carbon powder and titanium dioxide, with absorption spectra shown in Figure 2b. Detailed fabrication methods and validation of phantom optical properties are described in our previous work.^30^ To make the solid phantom similar to biological tissue will require using a hemoglobin‐based phantom, which is very difficult to do because of the current regulation requirement concerning use of blood. Monte Carlo simulations were performed using the same optical properties as the phantoms, normalized at 665 nm to match the measured data (Figure 2b). The spectral differences between real tissue and our solid phantoms are known to influence the optical property dependence of total Cherenkov emission and will be discussed later.

These nine phantoms were irradiated under TSET conditions using 6 MeV high–dose–rate electrons (HDTSE) at an SSD of 500 cm and a gantry angle of 90°, with spoiler. Cherenkov emission was captured using a time‐gated intensified CMOS camera (DoseOptics LLC, Lebanon, NH), then processed by background subtraction, median filtering, and frame accumulation (Figure 2c). The mean Cherenkov intensity at the center of each phantom was plotted as a function of monitor units (MU), and linear fits were used to determine the slopes (Figure 2d). The resulting slopes were normalized to phantom 5 (µ_a_ = 0.4 cm^−^^1^, µ_s_′ = 10.88 cm^−^^1^, approximately the mean optical properties) to determine the measured normalized Cherenkov emission per dose.

## 3 RESULTS

**FIGURE 3:**
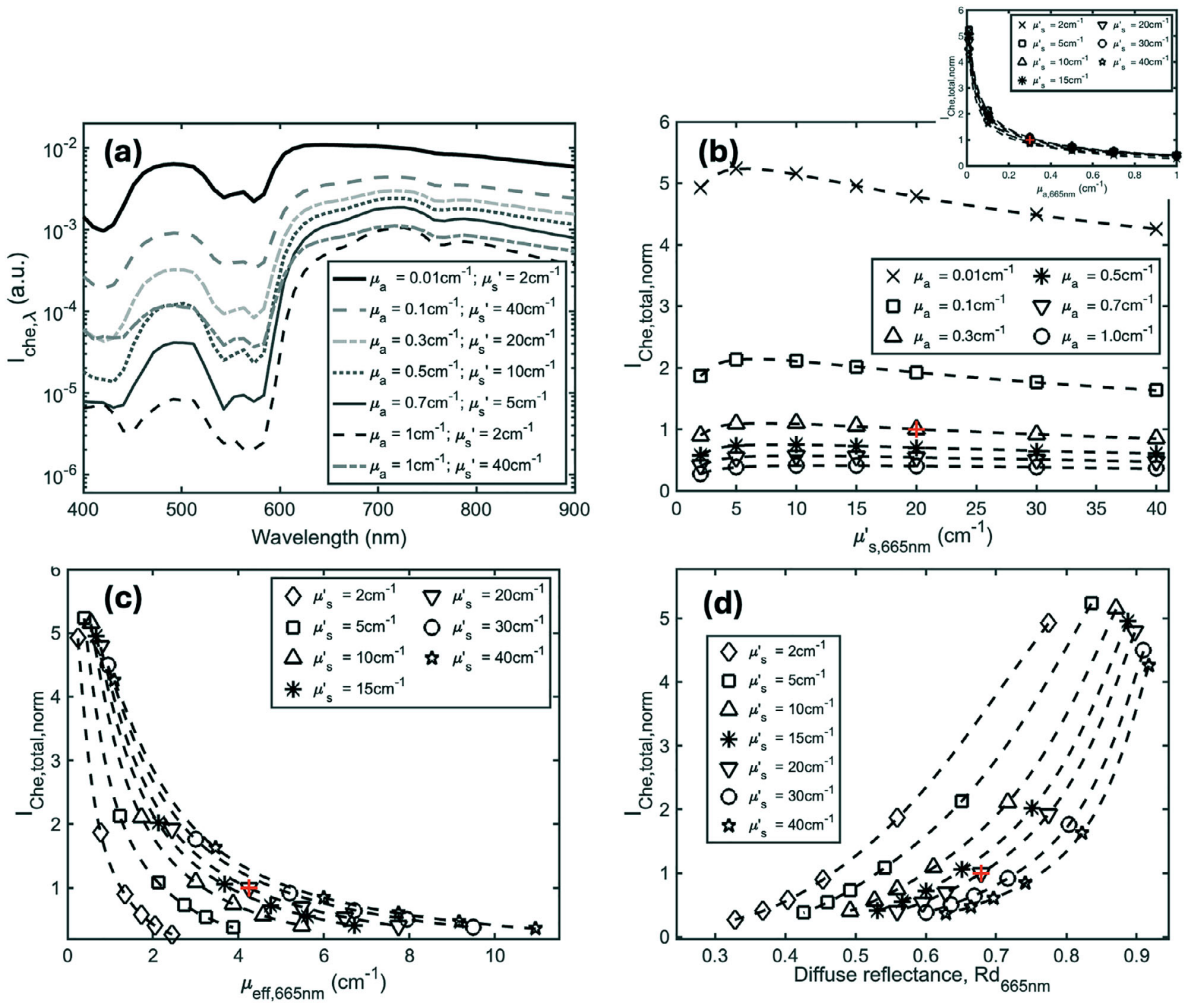
(a) Simulated Cherenkov emission spectra at 7 different tissue optical properties. Total Cherenkov emission (area under curve in (a)) as a function of (b) reduced scattering coefficient, (c) effective attenuation coefficient, and (d) diffuse reflectance at 665 nm. Values in (b)—(d) are normalized to 1 at µ_a,665 nm_ = 0.3 cm^−1^ and µ_s_, _665nm_ ' = 20 cm^−1^ (marked by the red “+” in the figures). In (b)—(d), Symbols are MC simulation and dashed line is the analytical expression (Equation 3). The insert in Figure 3b shows the normalized total Cherenkov emission versus absorption coefficient.

Figure 3(a) shows representative Cherenkov emission spectra (*I_Che, λ_*) simulated by MC modeling. *I_Che, λ_* was obtained by integrating C_em_ over exiting angle and radius, i.e. $I_{\mathrm{Che},\lambda }=\underset{0}{\int^{2\pi }}\underset{0}{\int^{\infty }}C_{\mathrm{em}}\mathrm{rd}r_{e}d\theta_{e}$. To show a clear figure, we have chosen 7 conditions covering the least absorbent and scattering conditions (*µ*_a_ = 0.01 and *µ*_s_’ = 2 cm^−1^) and the most absorbent and scattering conditions (*µ*_a_ = 1 and *µ*_s_’ = 40 cm^−1^). We arbitrarily choose 5 other conditions for µ_a_ and µ_s_’ in between these two extremes yet still show differentiable lines. Despite being primarily produced in the ultraviolet and blue regions, Cherenkov light emerging from the tissue surface is mainly composed of deeper‐penetrating red photons, while wavelengths below 600 nm are strongly attenuated by oxy‐ and deoxyhemoglobin absorption. As shown in Figure 3(a), Cherenkov emission intensity is maximal within the “biological window” (600–800 nm), peaking at 700 nm and diminishes at longer wavelengths following the λ^−^^2^ dependence. Tissue optical properties have huge impact on the overall intensity of Cherenkov emission. To systematically evaluate the effect of tissue optical properties on the total Cherenkov emission, we computed the total Cherenkov intensity (*I_Che,total_*) for each tissue optical properties by integrating *I_Che, λ_* over 400 nm‐900 nm wavelength range, i.e. $I_{\mathrm{Che},\mathrm{total}}=\underset{400\mathrm{nm}}{\int^{900\mathrm{nm}}}I_{\mathrm{Che},\lambda }d\lambda$. The standard (maximum) percentage statistical uncertainty of the MC simulation of *I_che, total_* was 0.14% (0.23%) (see Table 1). *I_Che,total,norm_* is then normalized to 1 at tissue optical properties µ_a,665 nm_ = 0.3 cm^−1^ and µ_s_’_,665 nm_ = 20 cm^−1^, which is the mean measured patient's skin data.^34^ (Our measurement of mean skin tissue optical properties at 665 nm is the only published data^34^ we can find, although it was measured in patients undergoing HPPH‐mediated photodynamic therapy (PDT). The skin tissue optical properties near abdomen should not be affected by the administration of HPPH photosensitizers because hemoglobin absorption dominates at these wavelength range (550 – 700 nm).) Normalized *I_Che,total, norm_* was plotted against reduced scattering and absorption coefficients, effective attenuation coefficient (*µ_eff_*) and diffuse reflectance (R_d_) at 665 nm as shown in Figure 3b–d. Analytical expression of *µ_eff_* ^24^ and R_d_^24^ for air‐tissue interface (n_tissue _= 1.4) is given as 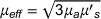 and $R_{d}=0.4843a^{\prime }\cdot \left(1+e^{-4.428\sqrt{1-a^{\prime }}}\right)\cdot e^{-2.65\sqrt{1-a^{\prime }}}$, where $a^{\prime }=\frac{\mu_{s}^{\prime }}{\mu_{a}+\mu_{s}^{\prime }}$, respectively.

Figures 3b–d show two‐dimensional dependence of Cherenkov intensity to tissue reduced scattering coefficients, absorption coefficients (in Figure 3b insert), effective attenuation coefficients, and diffuse reflective coefficients, respectively. Results show that Cherenkov intensity decreases with increasing tissue absorption and effective attenuation coefficients but increases then decreases with tissue scattering coefficients. Based on a fit to MC results in the tissue optical properties range show in Table 1, a two‐dimensional (µ_a_ and µ_s_’) dependence of (unnormalized) Cherenkov intensity can be expressed analytically as:

(3)

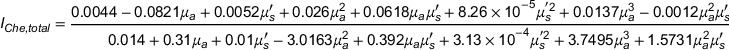




This analytical expression (Equation 3) agrees with Monte Carlo simulation to within standard (maximum) percentage deviation of 0.6% (1.8%) (see Table 2), allowing convenient calculation of Cherenkov intensity for any tissue absorption and reduced scattering coefficients at 665 nm.

**TABLE 2:** Percentage (%) deviation of total Cherenkov emission IChe, total between Monte Carlo simulation and analytical function (Equation 3). The std for the entire phantom $\sqrt{\sum \sigma^{2}/\left(n-1\right)}$ is 0.61%.

µ_a_ / µ_s_'	2 cm^−1^	5 cm^−1^	10 cm^−1^	15 cm^−1^	20 cm^−1^	30 cm^−1^	40 cm^−1^
0.01 cm^−1^	0.1	0.2	0.1	0.5	0.1	0.0	0.4
0.1 cm^−1^	0.0	0.3	0.0	0.3	0.4	0.0	0.2
0.3 cm^−1^	0.4	−0.2	−0.8	−0.8	0.4	0.7	−0.2
0.5 cm^−1^	0.1	0.7	−0.4	0.8	1.0	1.5	−0.1
0.7 cm^−1^	−1.0	0.1	0.1	0.8	1.3	0.3	−0.4
1 cm^−1^	1.0	0.2	−0.9	0.0	−0.3	−0.1	−1.8

**FIGURE 4:**
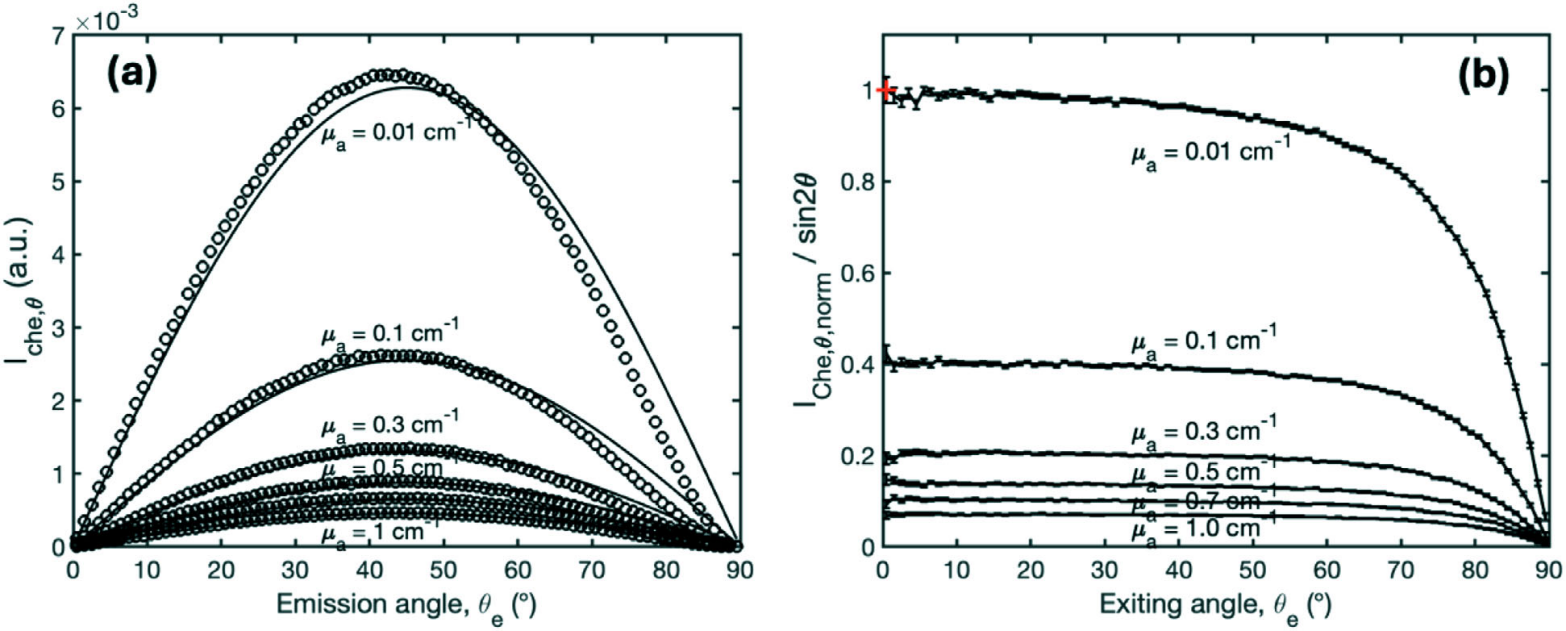
(a) Angular distribution of Cherenkov emission as a function of reduced scattering coefficient at 665 nm, µ_s,665nm_' = 5 cm^−1^, and absorption coefficient at 665 nm, µ_a,665_ _nm_ (from top to bottom) = 0.01 cm^−1^, 0.1 cm^−1^, 0.3 cm^−1^, 0.5 cm^−1^, 0.7 cm^−1^, and 1.0 cm^−1^. Theoretical Lambertian distribution function sin2θ is plotted and overlay on each simulation. I_Che,θ_ is defined as $I_{\mathrm{Che},\theta }=\underset{400\mathrm{nm}}{\int^{900\mathrm{nm}}}\underset{0}{\int^{\infty }}C_{\mathrm{em}}\mathrm{rd}r_{e}d\lambda$. (b) I_che,θ_ _, norm_/sin2θ normalized to be 1 at θ = 0° for optical properties µ_a,665 nm_ = 0.01 cm^−1^ and µ_s,__665nm_' = 5 cm^−1^(marked by the red “+” in the figures).

Figure 4a shows the angular distribution of Cherenkov emission at the tissue surface under different optical properties. *I_Che,θ_* was computed by integrating *C_em_* over wavelength and exiting radius, i.e. $I_{\mathrm{Che},\theta }=\underset{400\mathrm{nm}}{\int^{900\mathrm{nm}}}\underset{0}{\int^{\infty }}C_{\mathrm{em}}\mathrm{rd}r_{e}d\lambda$. From photon conservation, diffuse reflectance R_d_ can be expressed in terms of radiance *L* as $R_{d}.d\theta =L\cdot d\Omega /\phi_{\mathrm{air}}$, where *ϕ*_air_ is the incident fluence rate, and *dΩ *= *2πsinθdθ*, which yields $L=R_{d}\cdot \phi_{\mathrm{air}}/\left(2\pi \sin\theta \right)$. As shown in Figure 4a, the angular dependence of the MC calculated *I_Che,θ_* can be generally described by *sin2θ = 2sinθcosθ* for all simulated optical properties. We have decided to vary µ_a_ instead of µ_s_’ in Figure 4a because otherwise all the curves will be overlap on top of each other. However, when the function *sin2θ* (solid line) was overlayed on the simulation results (circles), the Cherenkov emission was found to deviate from this distribution at exit angles greater than 60°. To further examine the angular distribution of Cherenkov emission, we divide *I_Che,θ_* (for µ_a,665 nm_ = 0.01 ‐ 1 cm^−1^ and µ_s_’_,665 nm_ = 5 cm^−1^) by *sin2θ*. Then *I_Che,θ_* /*sin2θ* was normalizing to 1 at 0° exiting angle for µ_a,665 nm_ = 0.01 cm^−1^ as shown in Figure 4b. Cherenkov intensity obeys the Lambertian distribution for small exiting angles up to 60° as it stays relatively constant to within ± 5% uncertainty but rapidly falls off at larger exiting angles when photons exit close to parallel to the tissue surface.

**FIGURE 5:**
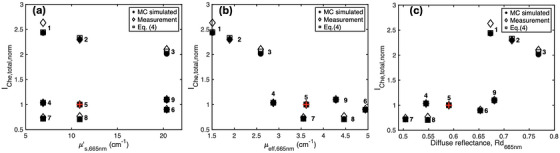
Normalized total Cherenkov emission, I_che, total, norm_, as a function of (a) reduced scattering coefficient, (b) effective attenuation coefficient at 665 nm, and (c) diffuse reflectance at 665 nm. Values in (a)—(c) are normalized to 1 at µ_a,665 nm_ = 0.4 cm^−1^ and µ_s,__665nm_' = 10.88 cm^−1^ (phantom 5, marked by the red “+” in the figures). Symbols used: Diamonds are the measurement, Circles are in‐house MC simulation, Squares are Equation (4) (an analytical fit to MC).

To validate tissue optical properties dependence, we have performed experiments on a set of solid phantoms with various tissue optical properties (see Figure 2a). These solid phantoms are made of a mixture of Silicone gel Carbon (µ_a_ in Figure 2b) and similar reduced scattering coefficient as shown in Figure 1c. Tissue optical properties at 665 nm are independently characterized using a dual‐catheter method.^35^ Figures 5a–c show the dependence of normalized total Cherenkov emission, *I_Che, total_*_, norm_, normalized to tissue optical properties (µ_a_ = 0.4 cm^−1^, µ_s_’ = 10.88 cm^−1^, phantom 5, which is the closest to the average µ_a_ and µ_s_’ among all nine phantoms), on reduced scattering coefficient, effective attenuation coefficient, and diffuse reflectance, respectively. The open diamonds are measured results, the closed circles are the Monte Carlo simulations using the absorption spectrum shown in Figure 2b, and the open squares are calculation based on Equation 4. Equation 4 is determined by fitting an analytical rational function to inhouse MC calculated total Cherenkov emissions for the experimental phantoms using the µ_a_ and µ_s_’ at 665 nm (µ_a_ = 0.05, 0.1, 0.2, 0.3, 0.4, 0.5, 0.6, 0.7 cm^−1^, µ_s_’ = 5, 8, 10, 12, 15, 20, 25 cm^−1^):

(4)

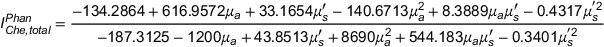




Equation 4 fits the corresponding MC results for the solid phantom to a standard (maximum) uncertainty of 0.6% (1.0%) for the range of tissue optical properties of µ_a_ = 0.05 – 0.7 cm^−1^ and µ_s_’ = 5 – 25 cm^−1^. Figure 5a–c show agreements between Equation (4) and the MC simulation for the optical properties used in the tissue‐simulating phantom experiments. Table 3 shows the comparison in more details. The standard (maximum) percentage deviation between inhouse MC and experiment are 2.8% (7.6%). We have run the MC simulation using Carbon spectrum to determine the total Cherenkov emission for optical property conditions at 665 nm.

**TABLE 3:** Optical properties of the solid phantom at 665 nm used in the experiment and the results of the normalized Cherenkov emission per dose from measurement and in‐house Monte Carlo code. (Bold optical properties, approximately the mean optical properties, are the reference condition for normalization.).

Index	µ_a,665 nm_ (cm^−1^)	µ_s, 665nm_ (cm^−1^)	In‐house MC	Expt	Diff (%)
1	0.11	6.89	2.430	2.632	−7.6
2	0.11	10.88	2.301	2.299	0.0
3	0.11	20.35	2.013	2.103	−4.3
4	0.4	6.89	1.036	1.034	0.1
5	**0.4**	**10.88**	1.000	1.000	0.0
6	0.4	20.35	0.890	0.906	−1.8
7	0.61	6.89	0.721	0.743	−3.0
8	0.61	10.88	0.711	0.765	−7.1
9	0.30	20.35	1.087	1.103	−1.5
				Average	2.8

**FIGURE 6:**
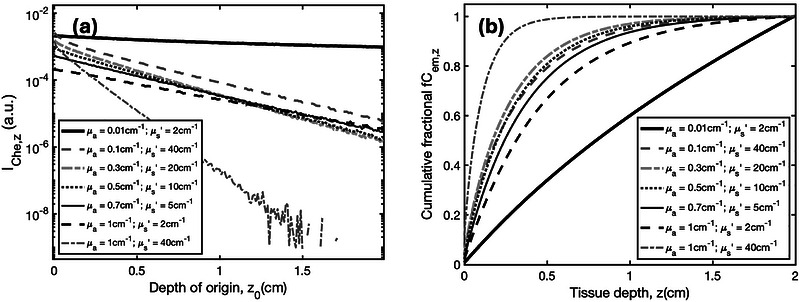
The initial depths of Cherenkov photon source are uniformly distributed between 0 and 2 cm to investigate the originated depth of all emission photons. (a) Depth of origin distribution of Cherenkov emission in tissues with seven different optical properties. I_Che,z_ is defined as $I_{\mathrm{Che},z}=\underset{400\mathrm{nm}}{\int^{900\mathrm{nm}}}\underset{0}{\int^{2\pi }}\underset{0}{\int^{\infty }}C_{\mathrm{em}}\mathrm{rd}r_{e}d\theta_{e}d\lambda$. (b) Cumulative fractional Cherenkov emission as a function of tissue depth and optical properties.

Next, we examined the depth of origin distribution of surface detected Cherenkov photons by plotting the *I_Che,z_* as a function of *z_0._* The depth of the initial Cherenkov photons was extended to 2 cm, corresponding to the electrons randomly distributed from 0–2 cm. Figure 6a shows that, the number of the Cherenkov photons detected at the surface decreased exponentially with increasing depth of origin, with the slope proportional to effective attenuation coefficient of the corresponding tissue optical properties, and were mainly from superficial tissue layers for the investigated optical properties. To evaluate the depth contribution of Cherenkov emitted at the surface under varying optical properties, we scored the cumulative fractional Cherenkov intensity, *fC_em,z_* as a function of tissue depth as shown in Figure 6b. Cumulative fractional Cherenkov intensity is defined as $fC_{\mathrm{em},z_{i}}=\underset{0}{\int^{z_{i}}}C_{\mathrm{em},z}\mathrm{dz}/\underset{0}{\int^{2\mathrm{cm}}}C_{\mathrm{em},z}\mathrm{dz}$, where *z_i _* = 0, 0.01, 0.02 … 2 cm. With decreasing reduced scattering and absorption, the contribution of Cherenkov emission from deeper tissue layers becomes more significant. Much of the Cherenkov emission at the surface are originated from the superficial layers as indicated by the steep rise of the curves in Figure 6b, which then level off into a plateau at different depths depending on tissue optical properties. Across all simulated optical properties, 80% of the surface Cherenkov emission originated from superficial tissue layers between 0.17 and 2.0 cm in depth, with a mean thickness of 0.72 cm. Table 4 summarizes the superficial layer thickness contributing 80% of the total Cherenkov emission detected at the surface. Note, the Monte Carlo study for depth dependence of Cherenkov emission uses Cherenkov photons uniformly distributed between 0 and 2 cm, instead of 0 – 1 cm used for the rest of the paper, to ensure Cherenkov photons generated at sufficiently deeper depths are included. Cherenkov photons are unlikely to exist at depths deeper than 2 cm for 3.84 MeV electron beam, which has a practical range of 1.9 cm. We have set the thickness of superficial tissue layers that contributes 80% of surface Cherenkov emission to 2.0 cm, the limit of MC simulation, (see Table 4 for data marked with *) for conditions where the optical penetration depth (*l* = 1/µ_eff_, 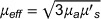) is larger than the calculated 80% of surface Cherenkov emission because this is indicative that the curve shape is still linear and will not bend over before it reaches 2.0 cm, the limit of MC simulation (see Figure 6b, solid curve for µ_a_ = 0.01 cm^−1^, µ_s_’ = 2 cm^−1^).

**TABLE 4:** Thickness of superficial tissue layers (cm) that contributes 80% of surface Cherenkov emission for tissue optical properties µ_a,665 nm_ = 0.01–1 cm^−1^ and µ_s,__665nm_' = 2–40 cm^−1^. Unit: cm. The mean tissue thickness of Table 4 is 0.72 cm. (Note, the Monte Carlo study uses Cherenkov photon sources uniformly distributed between 0 and 2 cm for this study to ensure we examine Cherenkov photons generated at sufficiently deeper depths. However, it is unlikely to have any Cherenkov photons generated deeper than 2 cm for 3.84 MeV electron beam, which has a practical range of 1.9 cm.).

µ_a_ / µ_s_'	2 cm^−1^	5 cm^−1^	10 cm^−1^	15 cm^−1^	20 cm^−1^	30 cm^−1^	40 cm^−1^
0.01 cm^−1^	2.00^*^	2.00^*^	2.00^*^	2.00^*^	1.23	1.16	1.10
0.1 cm^−1^	1.31	1.13	0.93	0.81	0.72	0.59	0.52
0.3 cm^−1^	1.11	0.84	0.62	0.51	0.44	0.35	0.30
0.5 cm^−1^	0.96	0.68	0.49	0.40	0.34	0.28	0.24
0.7 cm^−1^	0.85	0.58	0.42	0.34	0.29	0.23	0.20
1 cm^−1^	0.73	0.49	0.35	0.28	0.24	0.19	0.17

* We have set the superficial tissue layers that contribute 80% of superficial Cherenkov emission to be 2.0 cm, the limit of MC simulation, because the calculated values are less than the optical penetration depth for the tissue optical properties at 665 nm (*l_opt _* = 1/µ_eff _= 4.1, 2.58, 1.83, 1.49 cm for µ_a_ = 0.01 cm^−1^, and µ_s_’ = 2, 5, 10, 15 cm^−1^, respectively, where 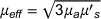).

**FIGURE 7:**
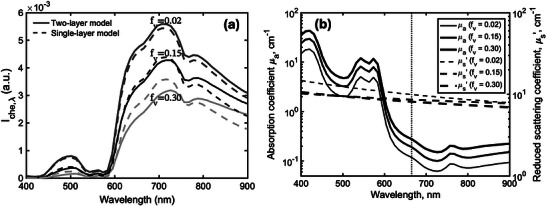
(a) Cherenkov emission spectra predicted by the direct two‐layer skin model (solid lines) and the effective bulk approximation (dashed lines) for melanosome volume fractions *f_v_* = 0.02, 0.15, and 0.30. The two‐layer model consists of a 100 µm epidermal melanin layer over underlying tissue with µ_a,665 nm_ = 0.1 cm^−1^ and µ_s,665nm_' = 10 cm^−1^. The normalized total Cherenkov emissions for the two‐layer (one‐layer) models are I_Che, total, norm_ = 2.016 (1.929), 1.536 (1.476), 1.144 (1.159). (b) The bulk tissue optical properties (µ_a_ and µ_s_’) for the single layer were obtained by fitting the diffuse reflectance spectrum (DRS) of the two‐layer model with melanin using a spectral fitting framework for two components (oxy‐ and deoxy‐ hemoglobin), see text for details. Solid lines represent the absorption coefficients (µ_a_), and dash‐dot lines represent the reduced scattering coefficients (µ_s_’).

To further evaluate the impact of superficial melanin absorption on the predicted Cherenkov emission, Figure 7a compares the Cherenkov emission spectra predicted by the direct two‐layer skin model and by the effective bulk approximation for melanosome volume fractions of *f_v_* = 0.02, 0.15, and 0.30, using an underlying tissue with µ_a,665nm_ = 0.1 cm^−1^, µ_s,665nm_’ = 10 cm^−1^. The total Cherenkov emission $I_{\mathrm{Che},\theta }$ are 0.136 (0.130), 0.104 (0.100), and 0.077 (0.078) for the three melanin conditions with the two‐layer (one‐layer) models. Based on the spectral component fit,^31^, ^34^ both absorption coefficient and reduced scattering coefficient for the single‐layer model were obtained (Figure 7b). The effective bulk µ_a_ at 665 nm for the single‐layer model was 0.12, 0.19 and 0.28 cm^−1^, respectively. Using these fitted values, the single‐layer model preserved the magnitude of the two‐layer model well, with differences in total Cherenkov emission of −4.3%, ‐3.9% and 1.3% respectively. The normalized total Cherenkov emissions for the two‐layer (one‐layer) models are *I_Che, total_*_, norm_ = 2.016 (1.929), 1.536 (1.476), 1.144 (1.159). It also reproduced the overall spectral trend, although deviations became more noticeable at higher melanin content. Overall, these results show that while superficial melanin alters the predicted Cherenkov emission, its effect can be captured to first order using a single‐layer model with bulk tissue optical properties.

## 4 DISCUSSIONS

In this study, we investigated the effect of tissue optical properties on the Cherenkov emission on tissue surface during TSET. A single layer semi‐infinite tissue model with homogenous absorption and reduced scattering coefficients was used in the simulations. Tissue absorption coefficient varies substantially over the visible spectrum due to the presence of tissue chromophores which absorb light differently in specific spectral range. These chromophores include hemoglobin, melanin and other proteins. As the contributions of other chromophores are relatively smaller in bulk tissue, hemoglobin is assumed to be the primary light absorber in the simulations, absorbing light dominantly in the UV‐blue region, where Cherenkov emission is the strongest. Hemoglobin exists in two forms, oxyhemoglobin (when oxygen molecule binds to hemoglobin) and deoxyhemoglobin (in the absence of oxygen molecules), each exhibit distinctive absorption spectra. The spectra of extinction coefficients for HbO_2_ and Hb used in this study were obtained from literature^20^ and a StO_2_ of 70% was used for which the ratio of [HbO2] to [Hb] was 70:30.^36^ While the tissue absorption coefficient varies greatly, the reduced scattering coefficient decreases monotonically with wavelength. The approximation *µ_s_’* (*λ*) = *A·λ^−b^* from the Mie theory has been shown to simulate tissue scattering reasonably well over our spectral range.^37^, ^38^

The simulated Cherenkov emission spectra in Figure 3 show that most detected Cherenkov photons on tissue surface are above 600 nm, with negligible contribution from the blue‐green region except for tissues with low absorption (0.01 cm^−1^) and high reduced scattering (40 cm^−1^). These results are in accordance with the Cherenkov emission spectra observed in optical phantom with hemoglobin, as well as those measured from in vivo experiments of tissue oxygenation.^39^ Unlike the characteristic brilliant blue of visible Cherenkov radiation observed in water tanks,^40^, ^41^ Cherenkov emission detected at tissue surface is expected to appear red color.^10^, ^42^

Cherenkov emissions are low visible light produced by interactions between high energy radiation and human tissues. The integral intensity of the emission spectrum is captured using specialized camera during real time Cherenkov imaging.^43^ Studies have shown a linear relationship between integrated Cherenkov intensity and radiation dose in water phantom with homogenous optical properties.^6^ However, inherent heterogeneity of tissue optical properties in human may distort the linearity between the Cherenkov signal and radiation dose and complicate the conversion of Cherenkov intensity to absorbed dose. Hachadorian et al.^42^ demonstrated correction of Cherenkov light attenuation in tissue for photon beams using tissue optical properties mapped by spatial frequency domain imaging and found that the effective attenuation and Cherenkov emission from a homogenous phantom follows an exponential decaying trend. In this study, we numerically and experimentally investigated the effect of tissue optical properties on integrated Cherenkov intensity and found a two‐dimensional dependence of Cherenkov intensity to tissue absorption and reduced scattering coefficients, as shown in Figure 3b. Unlike that reported by Hachadorian et al.,^42^ our MC simulated Cherenkov emission follows a two‐dimensional decay exponentially with effective attenuation coefficient, µ_eff_, and µ_s_’ as shown in Figure 3c rather than a single curve following µ_eff_, and increases as a two‐dimensional function of diffuse reflectance and µ_s_’ as shown in Figure 3d. We have found an analytical function (Equation 3) to fit the Monte Carlo results to within a standard (maximum) relative error of 0.6% (1.8%) (see Table 2).

When an electron travels at a speed (*v*) faster than the phase velocity of light in human tissue (*c/n*), Cherenkov light is emitted in a cone with half angle ζ along the direction of the moving electron; *c* is the speed of light in vacuum and *n* is the refractive index of human tissue. As Cherenkov photons are generated at a specific angle, which is related to speed of the traveling electron (*cos ζ =* *c/nv*), we systematically examined the angular distribution of the Cherenkov emission on surface for a wide range of tissue optical properties. Our simulated results suggest no preferential exiting angle of Cherenkov emission on tissue surface even though Cherenkov photons are generated at ζ = 44.0° in tissue. We investigated the distribution of initial depth of the Cherenkov emission and found that 80% of surface Cherenkov emission are generated from a superficial tissue layer of 0.17 cm to 2.0 cm for all tissue optical properties simulated in this study. These tissue depths are all greater than the photon mean‐free path *l = 1/(µ_a_+µ_s_’)* for each corresponding tissue optical properties. Therefore, the propagation direction of a Cherenkov photon is completely randomized before escaping from the surface. However, our results suggest Cherenkov emission follows the Lambertian distribution (*L* ∝ cos *θ*) when the exiting angle is smaller than 60° to the axis of air‐tissue interface (*I_Che,θ_* /*sin2θ* remains unchanged). When Cherenkov light is emitted at an angle approximately parallel to the tissue surface (or greater than 60°), it rapidly deviates from the analytical theory as demonstrated in Figure 4b.

Figure 5 shows that measured and MC simulated total normalized Cherenkov emission per dose for the nine phantoms agrees well to each other. The analytical equation (Equation 4) agrees well with the inhouse MC simulated normalized Cherenkov emission per dose. Total normalized Cherenkov emission for TSET electron beams does not follow a monotonic function to either effective attenuation coefficient, µ_eff_ (Figure 5b) or diffuse reflectance, R_d_ (Figure 5c), as has been suggested in the earlier literatures.^7^, ^42^

As shown in Figure 7, superficial melanin can substantially alter the predicted Cherenkov emission, but the effective single‐layer model reproduced the overall spectral trend and preserved the total emission to within a few percent for the conditions evaluated. These findings support the clinical relevance of the present framework because the diffuse reflectance spectroscopy measurement and spectral fitting methods were already used to estimate patient‐specific skin optical properties, which can then be incorporated into the interpretation of Cherenkov emission. Nevertheless, explicit multilayer and spatially heterogeneous tissue models would be more accurate for representing localized pigment variation and other complex absorber distributions, but they are beyond the scope of the present study.

**FIGURE 8:**
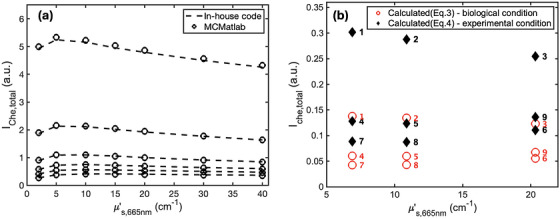
(a) Comparison of MC calculated total Cherenkov emission as a function of reduced scattering coefficient for MCmatlab and in‐house MC code for the same conditions as in Figure 3. (b) a comparison between calculation using Equation 4 based on fit to MC simulated total Cherenkov emission, I_Che, total_, for the experimental phantom using the measured spectrum for µ_a_ (Figure 2b), shown as black diamonds, and calculation using Equation 3 based on fit to MC simulation of biological spectrum for µ_a_ (Figure 1c) and rescaled to the same ma used in the experimental phantom at 665 nm, shown as red circles. The reduced scattering coefficient for both Equations. (3) and (4) following the Mie theory (Figure 1c) and rescaled to the same µ_s_’ used in the experimental phantom at 665 nm.

To validate our In‐house Monte Carlo code, we have compared the in‐house code with open‐source MC code (MCmatlab) for the total Cherenkov intensity, *I_Che, total_* for all ranges of optical properties of biological tissues in Table 1 and found them to agree with each other to within a standard (maximum) relative deviation of 1.7% (4.5%) (see Figure 8a). To emphasize the importance of the dependence of Cherenkov total emission vs. the absorption spectrum for the biological tissue (using blood hemoglobin spectrum, Figure 1c) vs. the solid phantom (using Carbon spectrum, Figure 2b), Figure 8b shows total Cherenkov emission between biological phantom (analytical calculated using Equation 3, open circles) and the experimental phantom (analytical calculated using Eq.4, filled diamonds) to be substantial different, even though the µ_a_ and µ_s_’ values at 665 nm are the same. Equation 3 accurately matches MC results only for biological tissues with spectrally varying hemoglobin absorption. It does not hold for constant absorption media (e.g., carbon as used in our experimental phantom) or other samples with different absorption spectrum even if µ_a_ and µ_s_’ at 665 nm are identical.

## 5 CONCLUSION

In this study, we studied tissue optical properties dependence of Cherenkov emission per delivered dose from a TSET electron beam in a semi‐infinite tissue geometry with homogenous optical properties and demonstrated that tissue optical properties can significantly affect the Cherenkov emission on tissue surface. Cherenkov emission decreases with increasing tissue absorption and changes minimally with tissue scattering. The optical properties dependence of Cherenkov emission can be expressed in two‐dimensional function of µ_a,665 nm_ and µ_s_’_,665 nm_. Cherenkov emission comes primarily from Cherenkov photons generated from superficial tissue layers. 80% of the surface Cherenkov emission originates from 0.17 cm to 2.0 cm of superficial tissue layers for the range of optical properties investigated in this study, with a mean tissue thickness of 0.72 cm in depth across the optical properties simulated (electron uniformly distributed along z = 0–2 cm) (see Table 4). Cherenkov emission follows the Lambertian distribution when Cherenkov photons exit within 60° to the axis of air‐tissue interface but rapidly deviates from the analytical theory when the Cherenkov photons exit approximately parallel to the tissue surface. Angle‐specific generation of Cherenkov radiation in tissue has not resulted in preferential exiting angle as the direction of propagation of most Cherenkov photons is randomized before escaping the surface.

## CONFLICT OF INTEREST STATEMENT

The authors declare no conflicts of interest.
